# The music that helps people sleep and the reasons they believe it works: A mixed methods analysis of online survey reports

**DOI:** 10.1371/journal.pone.0206531

**Published:** 2018-11-14

**Authors:** Tabitha Trahan, Simon J. Durrant, Daniel Müllensiefen, Victoria J. Williamson

**Affiliations:** 1 Department of Music, University of Sheffield, Sheffield, United Kingdom; 2 Department of Psychology, Goldsmiths, University of London, London, United Kingdom; 3 School of Psychology, University of Lincoln, Lincoln, United Kingdom; Neurocenter of Southern Switzerland, SWITZERLAND

## Abstract

Sleep loss is a widespread problem with serious physical and economic consequences. Music can impact upon physical, psychological and emotional states, which may explain anecdotal reports of its success as an everyday sleep aid. However, there is a lack of systematic data on how widely it is used, why people opt for music as a sleep aid, or what music works; hence the underlying drivers to music-sleep effects remain unclear. We investigated music as a sleep aid within the general public via a mixed methods data online survey (n = 651) that scored musicality, sleep habits, and open text responses on what music helps sleep and why. In total, 62% of respondents stated that they used music to help them sleep. They reported fourteen musical genres comprising 545 artists. Linear modelling found stress, age, and music use as significant predictors of sleep quality (PSQI) scores. Regression tree modelling revealed that younger people with higher musical engagement were significantly more likely to use music to aid sleep. Thematic analysis of the open text responses generated four themes that described why people believe music helps sleep: music offers unique properties that stimulate sleep (Provide), music is part of a normal sleep routine (Habit), music induces a physical or mental state conducive to sleep (State), and music blocks an internal or external stimulus that would otherwise disrupt sleep (Distract). This survey provides new evidence into the relationship between music and sleep in a population that ranged widely in age, musicality, sleep habits and stress levels. In particular, the results highlight the varied pathways of effect between music and sleep. Diversity was observed both in music choices, which reflected idiosyncratic preferences rather than any consistent musical structure, and in the reasons why music supports good sleep, which went far beyond simple physical/mental relaxation.

## Introduction

Thomas Dekker said, “Sleep is that golden chain that ties health and our bodies together” [1]. However, for many people in modern society, the chain is in danger of being broken as sleep problems become ever more prevalent. Around 40% of United Kingdom adults suffer from disrupted sleep [2], a trend mirrored in the United States with approximately 50–70 million American adults reporting sleep difficulties [3]. Sleep loss has been linked to a range of physical and mental health issues, with short-term effects evident after a single night of poor sleep. Short-term memory may be impaired [4] and participants report lower levels of happiness and more feelings of depression [5]. Work-related and driving accident rates are also thought to increase as a result of reduced cognitive speed and efficiency [6]. If short term sleep loss is not remedied there is the risk that it may become chronic, a situation associated with serious health and wellbeing challenges [7–9].

Attempts to remedy poor sleep include the pervasive use of pharmaceutical sleep aids, which come with a list of negative side effects. Sales of pharmaceuticals have risen steadily, up 31% in the UK between 2006 and 2011 [10]. Pharmacies within the UK dispensed more than 15.2 million prescriptions for sleep aids during 2010–2011 [11] equating to around £50 million [12] or nearly 1 in 10 adults taking some form of pharmaceutical intervention [13] on a regular basis. In the United States, a survey suggested a 293% increase in the number of sleep related prescriptions from 5.3 to 20.8 million prescriptions from 1999 to 2010 [14]. Pharmaceutical sleep aids have been linked to negative side effects that increase with long-term use, including nausea, dizziness, dependency and withdrawal, amnesia, seizures, and even an increase in mortality [15,16]. Given the prevalence, cost, and potentially harmful side effects of pharmaceutical sleep aids, the search for low cost, non-pharmaceutical alternatives aid has become a priority.

Music has many promising neurological and physiological effects that may be indicative of its effective use in the fight against sleep loss. In some clinical populations listening to music has been suggested to reduce anxiety [17,18] and the subjectively negative effects of physical pain [19]. Potential mechanisms for effect are ascribed to the modulation of sympathetic nervous system activity [20] and levels of the stress hormone cortisol [18,21,22]. The subjective psychological benefits of music have also been linked to chemical changes observed via hormone levels. A study measuring self-reported relaxation in groups with different objective levels of oxytocin release found that music increased oxytocin and accordingly levels of relaxation compared to control groups [23]. Outside of clinical practice, music is frequently used to self-regulate mood and arousal [24,25] as well as to decrease negative thoughts [26,27]. Given the established links between stress and poor sleep, this research provides indicative evidence to suggest that music may be a powerful tool in the fight against sleep loss.

Studies into music’s efficacy as a sleep aid have used subjective self-report measures and occasionally objective measures such as actigraphy and polysomnography. The majority have been conducted in clinical populations such as individuals with chronic insomnia or patients in hospital settings [28–30]. For example, Chang et al. [28] demonstrated that listening to music for 45 minutes prior to sleep for four days shortened stage 2 sleep duration, while extending REM sleep in adults with chronic insomnia. Research by Chen et al. [31] supported these findings in a group of young adults. Individuals with a long sleep latency (10 minutes or longer) saw a shorter stage 2 sleep and a longer deep sleep with sedative music playing for the first hour the participant was in bed.

With a growing body of evidence of successful music interventions in clinical populations, a potential therapeutic benefit exists for populations coping with transient insomnia due to life circumstances. Support for this claim has come from a recent Cochrane report that reviewed the music for sleep literature and concluded that the daily use of music, prior to sleep, was effective in improving overall sleep quality [32]. However, it is important to note that not all research has shown music is an effective sleep aid. Lazic and Ogilvie [33] found no significant improvement in polysomnographic measures of sleep with music as compared to a tone and control group. This mixed level of agreement on music’s success in the face of anecdotal reports led us to ask on what basis and in what manner people may be using music intuitively to help them sleep. Such insights have the potential to guide both effective and ecologically valid designs of sleep studies in the laboratory.

While music appears to have potential to aid sleep, there is no systematic population-based evidence of how it is being used, either in terms of prevalence, music choices or reasons for choices. It seems likely that music with certain characteristics, such as slow, quiet and minimal modulation, may be more suitable for aiding sleep than other music and some musical genres (such as new age music) will embody these characteristics more than others. However, this has not been systematically investigated to date either experimentally or at a population level. The current study is a first attempt to rectify this situation and to build on the literature by investigating the effects of using music in a short-term, self-help environment. Our study consists of the first online survey into the use of music as a sleep aid in the general population, and comprises three main research questions: (1) Who is using music to aid them in the process of sleep? For example, are age, gender, or musical training or engagement important? (2) For those who are using music, what kind of music do they choose? (3) Why do people believe that using music helps to improve their sleep?

## Methods and materials

This research was carried out in strict accordance with the approval of the research ethics committees of Goldsmiths, University of London and The University of Sheffield and was conducted according to the principles expressed in the Declaration of Helsinki. All participants provided specific online consent for their participation and had the right to withdraw at any time with no penalty.

A substantial online survey was created in order to investigate the use of music as a sleep aid in a general population using the Qualtrics online survey platform [34]. Piloting was done to ensure questions were meaningful and clear. All members of the population over the age of 18 were invited to participate. Once launched, between 2014 and 2016, 651 individuals completed the survey (mean age = 33.41 years, SD = 12.41, range = 18–79; 67% female). Participants were recruited globally using online social media platforms (e.g. Twitter, Facebook), emails to international institutions that allow recruitment drives amongst staff/ students, and word of mouth from all four authors (based in the UK, US and Germany). The survey questions were distributed in English. An acceptable understanding of the English language was determined by the quality of the free text response questions. The majority of final participants (80.65%, n = 525) came from the UK.

No specifications regarding sleep habits or sleep efficiency were set during the recruitment period, no specific population groups were targeted and all responses (regardless of whether or not music was used during sleep) were included in the final data set. There was no attempt to recruit only music users at any point. All participants were entered into a prize draw for a £100 voucher as compensation for their involvement with the survey.

The survey comprised four self-report scales for background information, followed by custom-written mixed methods response questions. The self-report scales were designed to answer the question of who is using music to aid their sleep and how often they were using it, while the custom-written questions were designed to find out what type of music was being used and the reasons people were using music for this purpose.

The first two self-report scales used were the engagement and training subscales of the Goldsmiths Musical Sophistication Index (Gold-MSI), which is a validated and widely-used tool for measuring musical background and engagement [35]. The Active Engagement subscale has a theoretical score range of 9–63 with higher scores representing a higher level of musical engagement. Musical Training scores range from 7–49, again with higher scores denoting higher musical training. The third validated scale used was the Pittsburgh Sleep Quality Index (PSQI), which is again a widely-used and validated tool [36]. The 19 self-reported questions of the PSQI are grouped into 7 component scores of 0–3 each, resulting in a global score ranging from 0–21 points. A higher score indicates an overall poorer quality of sleep, with anything scoring greater than 5 on the PSQI scale being considered poor sleep quality. Finally, participants were asked to self-report their perceived stress during the last month (to match the time period of the PSQI) using a 0–10 slider control, with a higher score of self-perceived stress signifying a higher stress level.

**Fig 1:**
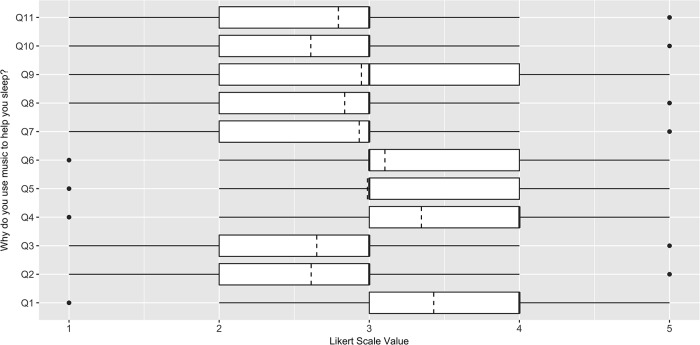
Responses to the limited option form from the UK sleep survey: “Why do you use music to help you sleep?” (n = 403). 5 = Strongly Agree, 4 = Agree, 3 = Neither Agree nor Disagree, 2 = Disagree, 1 = Strongly Disagree. Dashed line indicates the mean. Q1 = "Music helps me fall asleep sooner", Q2 = "Music reduces the number of times I wake up once I fall asleep", Q3 = "Music extends my total sleep time", Q4 = "Music reduces the amount of time I need to spend in bed before falling asleep", Q5 = "Music improves my sleep quality", Q6 = "Music increases my sleep satisfaction”, Q7 = "Music leads to deeper sleep", Q8 = "Music reduces my need for sleep medication", Q9 = "Music improves my dreams", Q10 = "Music helps reduce problem sleep behaviours (e.g. snoring, sleep walking, etc.)", Q11 = “Music helps me feel more refreshed on waking".

**Fig 2:**
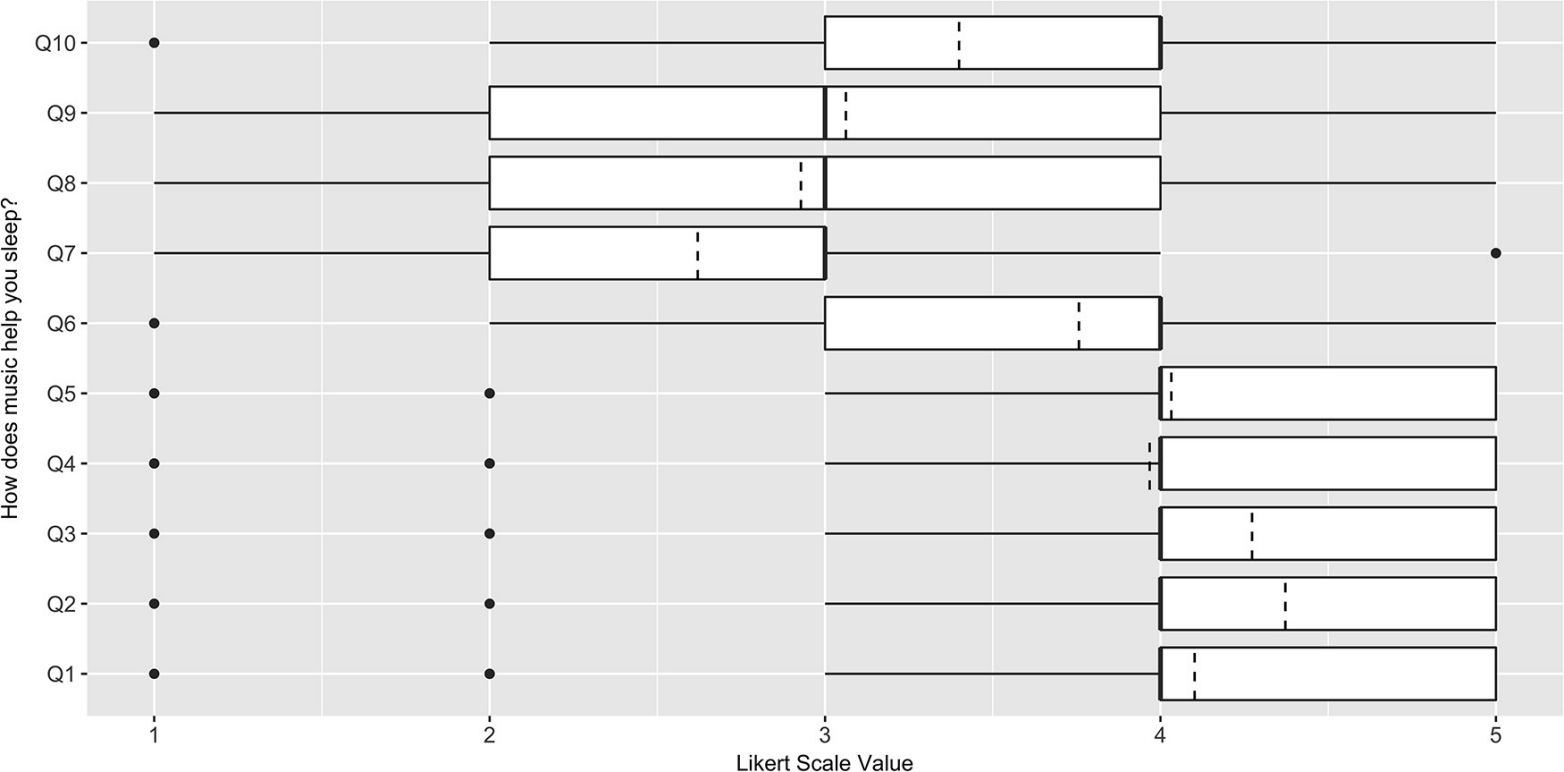
Responses to the limited option form from the UK sleep survey: “How do you use music to help you sleep?” (n = 403). Dashed line indicates the mean. Q1 = "Music helps me to physically relax", Q2 = "Music helps me to mentally relax", Q3 = "Music distracts me from the stress of the day just gone”, Q4 = "Music helps reduce worry about the next day", Q5 = "Music improves my mood before sleep", Q6 = "Music blocks out other sound that would otherwise distract my sleep", Q7 = "Music helps me to think about my day ahead”, Q8 = "Music helps me reflect on the day just past”, Q9 = "Music triggers memories that help me sleep", Q10 = "Music helps me to enter an alternative state/to meditate".

In order to address the research questions regarding the type of music chosen to aid sleep and the reasons people believe it to be helpful, we recorded both quantitative and qualitative data using three queries: “Please tell us what kinds of music help you to sleep”, “Why do you believe that music aids your sleep?”, and “What are the ways in which you believe that music aids your sleep?” To collect quantitative data relating to these questions, participants were prompted for their level of agreement, on 7-point Likert scales, to statements that were derived from the literature as being possible responses [32]. For example, to clarify *why* music might help sleep, statements included “Music increases my sleep satisfaction” and “Music helps reduce problem sleep behaviors (e.g. snoring, sleep walking, etc.)” (Fig 1). Similarly, to look at *how* music helps sleep, statements included “Music improves my mood before sleep” and “Music helps me reflect on the day just past” (Fig 2).

To gain further insight, each of the questions included a final option for an open text response. Participants were asked to fill in text boxes if they felt that they had a response that was not covered by the example statements or because they wanted to provide extra information relating to one or more of their responses. Data provided in these sections was collected for suitable analysis. Participants who claimed to use music to aid sleep were asked the frequency of their use on a 1–7 point Likert scale, with 1 meaning “less than once a year” to 7 meaning “every day”; the specific categories can be seen in Results Table 1. If participants stated they had used music to help them sleep we gave them the opportunity to tell us *what* kind of music they chose. Participants were encouraged to use as much detail as possible, including artists, genres, song title and albums. We used the 23 genres outlined by the Short Test Of Music Preferences questionnaire (STOMP) as a standardized list of musical genres for the post-hoc categorization of the participant’s responses [37]. The responses are tabulated in the results section.

**Table 1:** Frequency of music use as a sleep aid. Data reflects the 403 individuals who reported using music as a sleep aid in our survey (62% of the sample).

Frequency	Number	Percentage
less than once a year	62	15.38%
once or twice a year	89	22.08%
once or twice a month	107	26.55%
once or twice a week	70	17.37%
more than three times a week	25	6.2%
nearly every day	33	8.19%
every day	17	4.22%

All the quantitative data were subjected to statistical analysis using the party [38], ggplot2 [39], psych [40], and reshape2 [41], packages within R (version 3.4.0) [42]. Linear regression and tree-based models (random forest and classification trees from the R package party) were used to identify any meaningful relationships between sleep-related variables and other background variables (see detailed description in the results section). Open text responses, except those listing specific musical works or artists, were entered into a systematic qualitative thematic analysis technique based on Braun and Clarke [43] that was outlined in Williamson, Liikkanen, Jakubowski, & Stewart [44] and later developed in Alessandri et al. [45]. This form of theory-driven thematic analysis is a coding technique that prioritizes the minimization of subjective bias by setting out clear processes by which the analysis is to be conducted. The four-step process aims to reduce subjectivity at the points of both analysis and interpretation, by the requirement to have two independent ‘coders’ and a three stage process of theme development between them.

During the first stage of the process the two coders worked separately, line-by-line, to extract underlying themes within the written text. As each coder moved through the text they created new themes with appropriate definitions. These themes were used repeatedly throughout the analysis of the text, and new themes are created only if the text did not fit within the existing cohort. Once the coders had analyzed the entirety of the responses they came together for the first joint analysis. The second step of the process involved the coders taking alternating turns and systematically comparing each line of text. An agreement was reached as to the best theme labels and definitions to be utilized. These agreed upon themes and definitions were then organized into a ‘codebook’ and visual ‘code map’. The codebook comprises a written verbal account of the theme codes and their meanings used to describe the text by the smallest and most concise means. The code map is a visual representation of the relationships between these themes. For the third stage of the process, the coders independently applied these tools, codebook and code map, to the analysis of the whole of the text corpus for a second time. Following this re-analysis stage, the coders met for a second time to compare the distribution of the text into the agreed upon themes and discussed any difficulties with the codebook application. Coders were offered the opportunity to defend their selected themes if there was any disagreement. If an inter-rater disagreement remains, a third coder naïve to the disagreement made a final decision.

## Results

Descriptive statistics outlining the participants’ background information are presented in Table 2. The mean age of our sample was 33 years old (SD = 12.41) with the youngest participant being 18 years old and the oldest being 79 years old. Our sample had a mean score of 25.78 (SD = 12.34) on the GoldMSI Training subscale, which is higher than 46% of the general population on this test, slightly lower overall than the population mean. On the GoldMSI Engagement scale participants received a mean score of 37.22 (SD = 11.14), higher than 31% of the general population for this subscale, but lower than the population mean. The average PSQI score for our survey sample was 6.61 (SD = 3.43) which is slightly higher than averages for younger and older groups reported in past research [36,46,47]. Our sample’s self-reported mean stress score was 5.94 (SD = 2.49), and mean sleep efficiency was 84.10 (SD = 11.97).

**Table 2:** Descriptive statistics from the online sleep survey (n = 651; 32% male).

	Mean (percentile ranking)	SD	Median	Range
Age	33.41	12.41	31	18 to 79
GoldMSI-Training	25.78 (46)	12.34	27	7 to 49
GoldMSI-Engagement	37.22 (31)	11.14	38	9 to 62
PSQI	6.61	3.43	6	0 to 19
Stress	5.94	2.49	7	0 to 10
Sleep Efficiency	83.86	11.74	87.5	42.5 to 100

Out of the 651 respondents to the survey, 62% stated that they had used music (at least once) to help them sleep (n = 403). The descriptive statistics of these music users are outlined in Table 3, and of non-users in Table 4 and show that, on average, music users have a higher musicality, are younger, but also report higher level stress, a poorer sleep quality and are less sleep efficient. Combining these factors in a statistical model, a multiple regression was calculated with PSQI score predicted by stress, age, musical use and other demographic variables. Non-significant predictors were removed from the regression model. The final regression was significant (F(3,647) = 52.2, p < 0.001) with an R^2 of 0.1949 and an adjusted R^2 of 0.1912. The final model included music use (0.58, p = 0.022) (1 = use and 2 = no use), stress (0.57, p < 0.001), and age (0.033, p < 0.001) as significant predictor variables. This suggests that when age and stress increase and music is not used, the quality of sleep deteriorates, as indicated by an increasing PSQI scores.

**Table 3:** Descriptive statistics of participants from the online sleep survey that stated they had in the past used music as a sleep aid (n = 403; 31.27% male).

	Mean (percentile ranking)	SD	Median	Range
Age	31.97	11.60	29	18 to 74
GoldMSI-Training	26.35 (47)	12.15	28	7 to 49
GoldMSI-Engagement	38.94 (36)	10.07	39	9 to 62
PSQI	6.82	3.36	6	0 to 19
Stress	6.00	2.42	7	0 to 10
Sleep Efficiency	83.18	11.48	85.71	42.5 to 100

**Table 4:** Descriptive statistics of participants from the online sleep survey that stated they had not used music as a sleep aid (n = 248; 33.07% male).

	Mean (percentile ranking)	SD	Median	Range
Age	35.75	13.32	32	18 to 79
GoldMSI-Training	24.85 (42)	12.60	25	7 to 47
GoldMSI-Engagement	34.43 (23)	12.18	34	9 to 62
PSQI	6.29	3.53	5.5	0 to 19
Stress	5.85	2.61	7	0 to 10
Sleep Efficiency	84.95	12.09	86.28	44.44 to 100

One of our questions for the music users focused on the music they selected to aid sleep. Thematic classification was applied to the first open text question “You stated that you have in the past used music to help you sleep. Please tell us what kind of music helps you to sleep. Please provide as much detail as you can, including artists and albums.” The analysis involved organizing the text into identifiable mentions of known genres and artists using the 23 genres from the STOMP [37]. Genre groups that were mentioned within our survey and not represented by a STOMP genre were added to the genre battery. There were six new genres extracted from the survey: Acoustic, Ambient, Instrumental, Indie, Meditation, and House. There were 388 mentions of genre within the text from which we were able to classify 14 unique genres as depicted in Table 5. In total, 545 different artists were named within the survey. Johann Sebastian Bach was the most mentioned artist (N = 15) followed by Ed Sheeran (N = 13) and Wolfgang Amadeus Mozart (N = 13), Brian Eno (N = 10) and finally, Coldplay and Frédéric Chopin (both N = 9).

**Table 5:** Genres named in open response text, as a raw count and as a proportion of the total count.

Genre	n	Proportion
Classical	124	31.96%
Rock	42	10.82%
Pop	29	7.47%
Acoustic	26	6.70%
Jazz	24	6.19%
Soundtracks (film/theatre)	23	5.93%
Ambient	23	5.93%
Folk	20	5.15%
Instrumental	19	4.90%
Indie	16	4.12%
Meditation	16	4.12%
Metal	13	3.35%
Electronic	10	2.58%
House	3	0.77%

**Fig 3:**
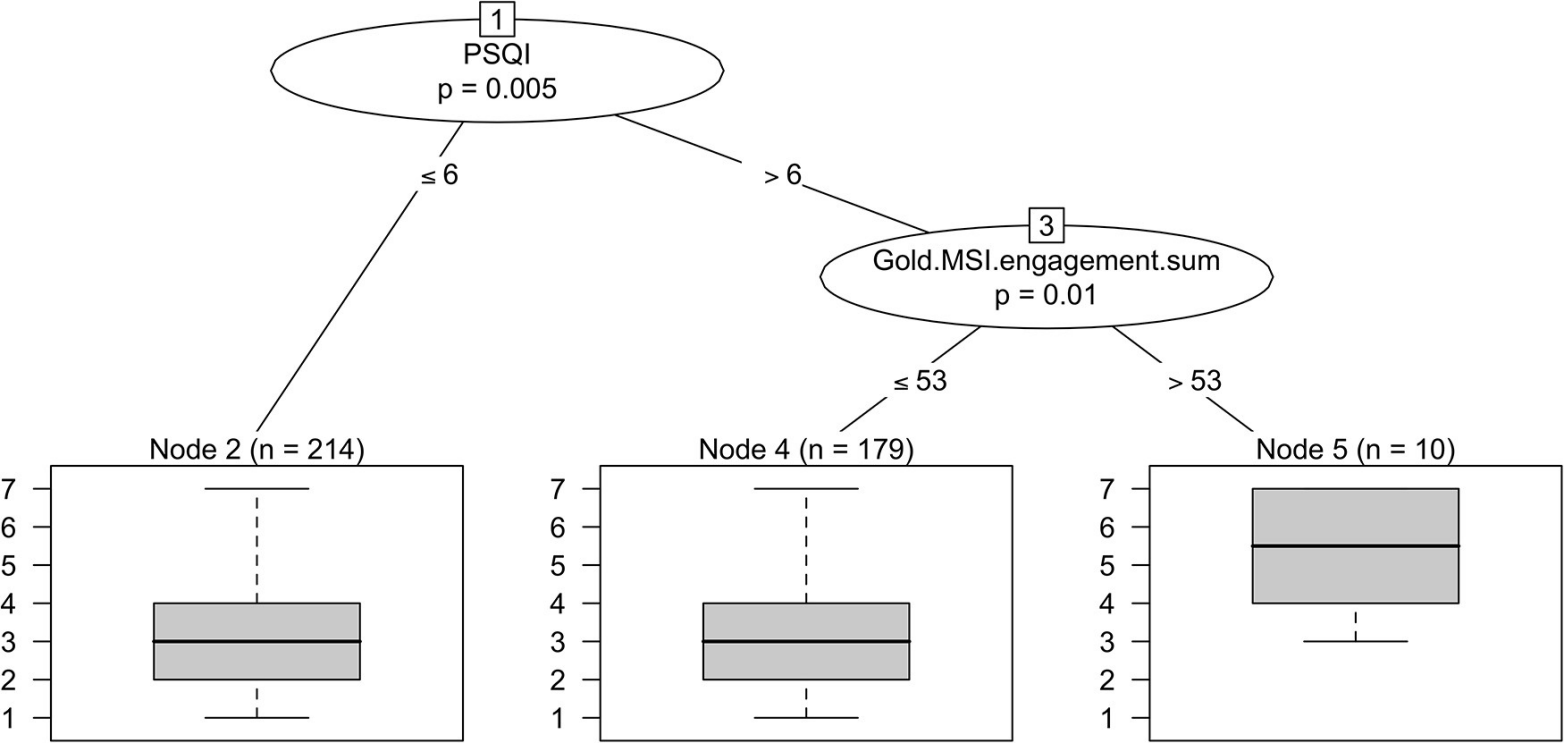
Regression tree predicting the frequency of music use as a sleep aid. The regression tree reveals a significant effect of PSQI and musical engagement on the frequency of music use as a sleep aid. Participants with a PSQI score greater than 6 (n = 189) and a Gold-MSI musical engagement score greater than 53 (n = 10) use music most frequently to help them sleep. This level of use of music as sleep aid is significantly higher than in the other two groups participants partitioned in this tree model: Participants with a PSQI score greater than 6 but a Gold-MSI musical engagement score equal to or less than 53 (n = 179) and participants with a PSQI score equal to or less than 6 (n = 214) use music less frequently to sleep. The variable ‘use of music as sleep aid’ is coded as follows: 1 = Less than once a year, 2 = Once–twice a year, 3 = Once–twice a month, 4 = Once–twice a week, 5 = Three or more times a week, 6 = Nearly everyday, 7 = Everyday.

We then looked at how often in general the 403 music users used music to help them sleep (Table 1). Overall, 35.98% (n = 145) claimed they used music at least weekly, with reports ranging from ‘once or twice a week’ (17.37%, n = 70) to ‘every day’ (4.22%, n = 17). The remaining participants claimed they used music less frequently. Using a regression tree we investigated what characteristics were predictive of the frequency of music use as a sleep aid as measured by the seven ordinal categories shown in Table 1; the results are shown in Fig 3. PSQI score and musical engagement as measured by the Gold-MSI were shown to be significant predictors of the frequency of music use in participants that claimed to use music to help them sleep. Specifically, participants with a PSQI score greater than 6 (n = 189) and a Gold-MSI musical engagement score greater than 53 (n = 10) use music most frequently to help them sleep.

**Fig 4:**
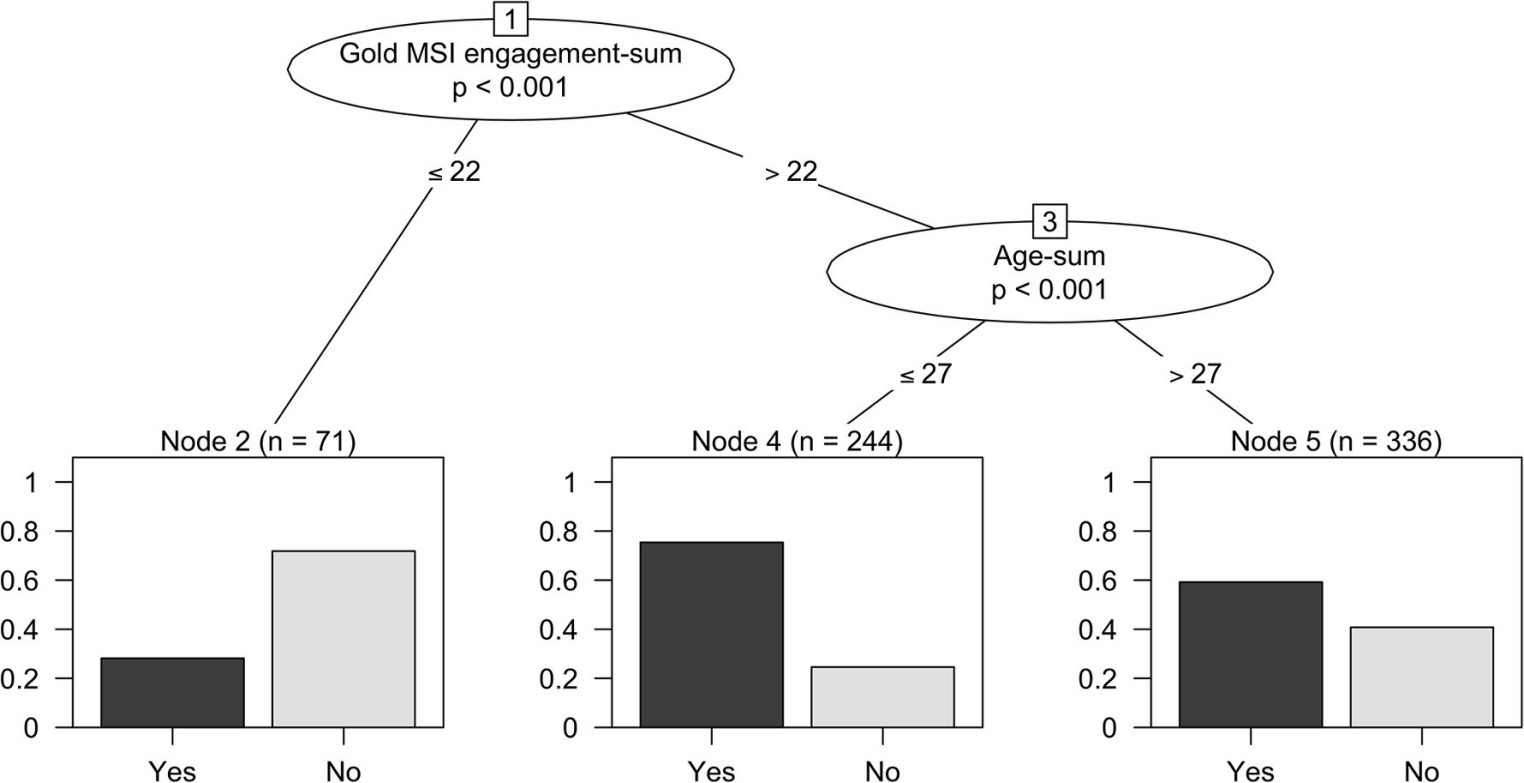
Classification tree for use of music as sleep aid. Each node represents the predicted proportion of participants using music as a sleep aid. Participants with low Gold MSI engagement scores (equal to or less than 22) fall in node 2, with a predicted 30% of participants of using music as a sleep aid. Participants with high Gold MSI engagement scores (> 22) are further split on age between nodes 4 and 5. Participants 27 years old and younger are more likely to use music (node 4, predicted proportion 75%) and those older than 27 are somewhat more likely to use music (node 5, predicted proportion 60%).

Classification and regression tree (CART) [38] modeling techniques were then utilized on the full set of 651 participants again, in order to investigate the individual factors that are associated with the use of music to help with sleep. These techniques map the effect of several predictors on a dependent variable by iteratively dividing observations into more and more homogeneous subgroups [48]. We computed a classification tree model with the binary dependent variable ‘use of music as sleep aid’, seven demographic predictor variables, PSQI, and the musical training and engagement scores from the Gold-MSI as independent variables. These seven demographic factors included were: age, gender, income, educational level, occupational area, current work status, and subjective stress level. The results of this model are shown in Fig 4. Both age and musical engagement were shown to be significantly associated with the propensity to use music as an aid to sleep. 70% of the people with a musical engagement score ≤ 22 indicated no regular use of music for sleep. In contrast, 75% of the people with a musical engagement score ≥ 22 and being 27 years old or younger reported using music to help them sleep.

In addition to these quantitative associations, we investigated *why* the 403 individuals in our sample use music to aid with sleep via both limited option statement and open response questions; the results from the limited option statement question of music users are summarized in Fig 1. The most common reason given for using music as a sleep aid was to ‘help fall asleep quicker’. 56.82% of participants who used music to help them sleep claimed they strongly agreed or agreed with this statement, and only 20.10% said they disagreed or strongly disagreed. This was followed by ‘reduction in time spent in bed before falling asleep’ (54.35%), and ‘increases sleep satisfaction’ (34.74%).

We also explored participant’s relative agreement levels with statements describing *how* music helped their sleep, these results are shown in Fig 2. The highest level of agreement was observed for the statement ‘Music helps me to mentally relax’, with 96.03% of the 403 participants who used music (n = 387) agreeing or strongly agreeing. This was followed by the statements ‘Music distracts me from the stress of the day just gone’ (91.81%) and ‘Music helps me to physically relax’ (85.85%).

**Fig 5:**
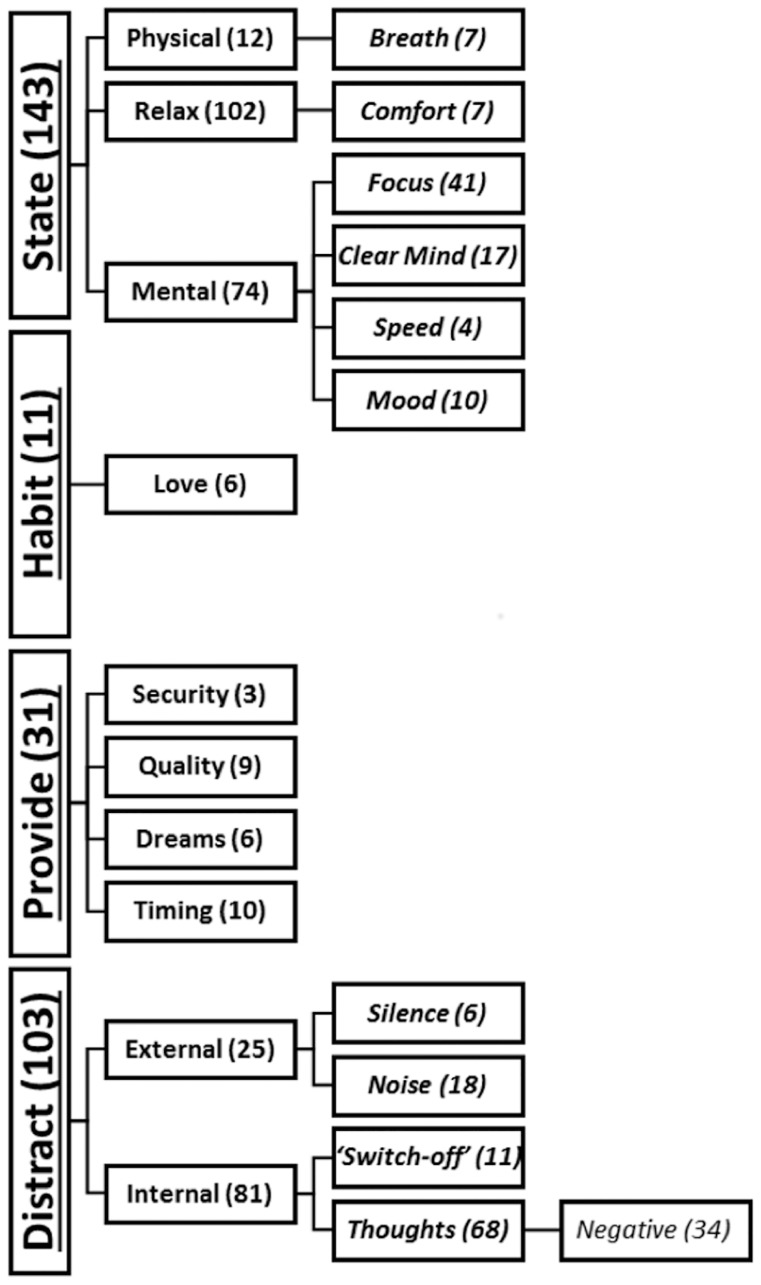
Visual theme map emergent from the sleep survey questions "How do you think music helps you sleep?". Visual map demonstrating the hierarchical organization of all themes and sub-themes. Counts of observed accounts for each theme are found in brackets next to the theme title.

The qualitative thematic analysis of these questions involved analyzing 219 responses. The open response text demonstrated an unclear distinction between the *how* and *why* prompts. We therefore aggregated the responses collected for “Please use this text box to tell us about any other reasons why you believe that music aids your sleep” and “Please use this text box to tell us about any other ways in which you believe that music aids your sleep”. The results of this combined analysis can be seen in Fig 5. This visual model demonstrates the main themes extracted from the text responses. The definitions for these themes are outlined below, along with sum totals for their frequency and text examples from the final codebook. The codebook developed during the thematic analysis of the online sleep survey open text responses to the question: “How does help you sleep?” consists of four hierarchical levels: **Level 1 Themes** written in bold and underlined text and followed by the frequency of coding in brackets, **Level 2 Themes** written in bold text, ***Level 3 Themes*** written in bold italicized text, and *Level 4 Themes* written in italicized text. In the case of each theme we provide one quote from the survey as an example. Level 1 Themes include the four primary themes that encompass all other motivations for music use during sleep: Distract, Provide, Habit, and State.

**State (143)**: This Level 1 theme outlined claims that the participant listens to music to change their state to one that they believe will boost their sleep in some way. The overall goal of this state shift resulted in three subsequent level 2 themes: mental, physical, or relaxation

- **Mental (74)**—Classifications of this level 2 theme were applied to comments in which the person aims to improve their mental state in advance of sleep with the use of music. This is comprised of four level 3 subthemes.
  - ***Focus (41)*** Describes instances where the person focuses their attention on the music itself or uses it to enter a state of generalized focus.
    “As a factor to focus the mind in something else “
  - ***Clear mind (17)*** Was specifically used if the person used the term ‘clear mind’ or its synonyms.
    “Music helps me clear my mind and fall asleep, and not notice the amount of time that it takes to do so.”
  - ***Speed (4)*** represents comments in which the participant refers to the speed of thoughts or mind; using speed related terms to describe cognitive processes.
    “It helps to calm the mind and reduce "racing thoughts”.”
  - ***Mood (10)*** Designated examples where a change in personal mood while listening to music or setting a desired environmental mood with the music was the goal.
    “It helps with my mood before falling asleep, which I think is a major factor in my ability to fall asleep.”
- **Relax (103)**–This level 2 theme contains cases where the participant used the term ‘relax’ or its synonyms. Additionally, any allusion to minimizing/combating stress and/or anxiety, this also includes claiming to be calmed or soothed.
  “It helped me feel more relaxed, and thus more likely to want to sleep.”
  - ***Comfort (7)*** More specifically, this level 3 theme of relax covers occasions where the person used the term ‘comfort’ or its synonyms to describe the way music makes them feel.
    “Makes me feel more comfortable.”
- **Physical (12)**—This time was used when a participant stated the aim was to improve their physical state in advance of sleep.
  “It works kind of like a lullaby—if the music is right, it can get me into a lovely sleepy state that makes it easier for my body to actually relax into sleep”
  - ***Breath (7)*** Further analysis isolated a single level 3 theme. This encompasses comments that point to music being used to regulate breathing; this also includes any mention of meditation practices.
    “Can match breathing to the music, which keeps it regular and slow (depending on the music) which stops anxiety and allows sleep to occur sooner. “

**Distract (103)**: This theme is defined as an attempt to block either a physical sound or a state of mind. This includes two level 2 themes related to external and internal distraction.

“Acts as a distraction when trying to fall asleep.”

- **External (25)**–Here the target of the distraction is external in the sense that the locus of the sound is outside the person’s body. This is further split into two level 3 subthemes.
  “It blocks out distracting noises while you’re trying to fall asleep.”
  - ***Silence*** *(6)* This theme points to the use of music to fill a void of external sound.
    “Background noise is comforting compared to complete silence.”
  - ***Noise (18)*** More commonly comments suggested the aim was to block noise.
    “It allows me to block out noises that stop me from sleeping e.g. clocks, snoring, fans.”
- **Internal (81)**–Utilized to describe instances when the target of the distraction is a subjective bodily or mental experience, this level 1 theme was made up of two level 2 subthemes.
  “It stops the busy chatter going round in my head before going to sleep.”
  - ***Switch off (11)*** Comments that explicitly used the term ‘switch off’ to refer to the experience were included here.
    “If I have a lot going round my head I think music is a good distraction which can help you switch off and fall asleep.”
  - ***Thoughts (68)*** Similarly, this theme was utilized when the person used the term ‘thoughts’ or its synonyms as the experience they wish to block.
    “It distracts me from thinking, which often prevents me from sleeping.”
    - *Negative (34)* If these thoughts were negative, worrisome, or stressful in nature they were placed in this level 3 theme.
      “Stops me thinking about unpleasant things.”

**Provide (31)**: For some people the use of music stimulates a secondary experience that facilitates sleep. We extrapolated four level 2 themes that described this process.

- **Timing (10)**–This expresses the use of music to help in the process of monitoring sleep time.
  “I find that it can help me track how long I’ve been asleep, and how long it took me to get to sleep.”
- **Security (3)**–In this level 2 theme comments were included if a real or imagined sense of security is felt because of music; it can include terms such as ‘warmth’, ‘safety’, and ‘company’.
  “I think it is reassuring that I am not going into complete subconscious emptiness and nothingness and the sound waves can comfort while I sleep.”
- **Dreams (6)**–Comments within this level 2 theme state that the music influences the individual’s dreams.
  “I find that music usually helps me sleep as day-dream while listening to music which usually turns into a dream.”
- **Quality (9)**–For some participants the music boosts an important aspect of sleep not covered in other codes at this level, including onset, depth or another aspect of the judged quality.
  “I feel the repetition is soothing. I believe this repetition translates during deep sleep. Perhaps rhythm aids in this process.”

**Habit (11)**: This Level 1 theme describes situations in which participants claim their motivation for using music is that they normally listen to music before sleep

“Though, I’ve done it for so long that it might just be habit.”

- **Love (6)**—For some this habit was born of the enjoyment of listening to music before sleep, as opposed to indifferent passive musical experiences.
  “I listen to music when going to bed not because it helps me sleep but because I want to listen to music since it is the only time of the day I can fully enjoy and focus on the music I listen to.”

## Discussion

It has been suggested that music can provide a low cost, non-pharmaceutical option for populations suffering from sleep difficulties [49]. The present study aimed to investigate this by gathering data from a general population recruited via self-selection online and through university advertisements without constraint. Our research questions were concerned with who is using music to help them sleep (including both demographics and musical background and engagement), what types of music they choose, and why people believe that music helps them to sleep.

The results of the survey support the hypothesis that many people who are not within a clinical environment or currently suffering from chronic insomnia (as identified by the PSQI standardized assessment instrument) are nevertheless using music in their everyday lives to help improve the quality of their sleep experiences. Our analysis indicated that music use was a significant predictor of PSQI score, with those using music less having higher PSQI scores, or lower sleep quality. It is notable that although our online survey focused on music for sleep, we found that only 62% of respondents reported using music for this purpose. This finding indicates that both music users and non-users chose to respond to the survey, and that although some response bias in favor of using music would be expected, our results cover a broad spectrum of participants. It is, however, impossible to know how representative our sample is in the absence of large-scale data obtained from different methods.

In terms of what these individuals chose to listen to within the music available to them, we note a large diversity within their responses, with great variety in the musical genres. This suggests support for the theory that self-selected music is more analgesic and anxiolytic in its effect than unfamiliar music [50], a finding that potentially diminishes the usefulness and efficacy of commercially available generic sleep playlists that consist of essentially sedative music. These playlists generally include tracks with relatively low tempo (60–80 beats per minute), low amplitude, and relatively little or slow-moving change, and are of a smooth/legato nature [51]. A next logical step for future research into the music that might best support sleep is to compare these two classes of music (self-selected and commercially available ‘sleep’ music) to better understand their similarities and differences, as well as their effectiveness both in the real world and under controlled lab conditions. An additional hypothesis that can be applied to such research, based on the findings of the present study, is that the desired effect of music use (i.e. the reason an individual believes music works for them) may play a role in the genre, artist, or song selected to aid in the sleep process. For example, the aim of regulating mood to improve sleep may require different genres, artists, and musical characteristics to those chosen when the aim is achieving distraction from negative thoughts or external sounds. On the other hand, it may be a combination of both the intended therapeutic use and the musical preference that optimizes music’s beneficial effect. These questions await experimental investigation.

Whilst the large diversity in music genres reported in the survey suggests that personal preference is important, we can’t rule out the potential for general musical features also to have an important role in developing targeted sleep music. The “perfect sleep song” may be one that compromises a number of baseline psychoacoustic guidelines that are then tailored to the individual to take into account their self-selection/preference. One example is the role of musical tempo, a basic property of music known to impact arousal levels [52]. In addition to potential ‘universal’ patterns of effect, interpersonal preferences for musical tempo are thought to be partly mediated by neural activity, especially in the motor cortex [53]. What is more, music preference has been shown to affect the experience of musical listening and this can alter the neurological responses of perception [54]. Conceivably, therefore, any future music for sleep interventions should reflect the complex relationship between the impacts of basic musical properties on brain and body responses, and personal preferences and prior familiarity.

In terms of the characteristics of the people who use music to help them sleep, the survey has indicated that musical experiences and perceptions are guided by differing qualities such as training [55], age and gender [56], and personality [37]. The Gold-MSI musical engagement scale has never before been utilized in the testing of a sleep intervention. To comprehend the degree to which music is having an effect on sleep, moderating factors such as musical engagement should be considered alongside music structure and preferences. A better understanding of the role of individual differences when it comes to using music as a sleep aid may also have implications for the function of music in wellbeing settings such as pain therapy and depression. In our data musical engagement played a significant role in the frequency of which individuals are using music as a sleep aid. The interaction of self-report musical engagement with participants’ PSQI scores was also predictive of the frequency of music use, with those of higher PSQI scores (i.e. poorer sleep) and higher musical engagement using music as a sleep aid more frequently. Hence in our sample, music provides an option for many who are seeking help at a low cost and with no determinable side effects. This study also suggests that music’s power in populations not necessarily suffering from comorbid ailments but who are simply seeking a good night’s rest As this survey cannot directly quantify the effect of music in its ability to aid in sleep, it is not possible from our data to confirm whether or not music objectively had a beneficial effect, but prior studies support the ongoing hypothesis that this is likely to be the case [28–30].

We also analyzed the outputs from the questions relating to *why* music helps people to sleep. The reasons stated for using music as a sleep aid were diverse in their origins and implications. The main reason people reported using music was as a tool to change one’s state of mind; whether to relax, focus or initiate a change in mood. This finding is in line with previous research which has suggested that music is used as part of everyday life to regulate mood [27] or reduce arousal [57,58] and anxiety [59]. It has been suggested that the effects of music on anxiety are biological [23]; music’s effect on sleep could well be mediated by these biological effects, and the autonomic systems for anxiety and arousal in particular. If these complex chemical and neural systems are being recruited by the use of music, it is reasonable to suggest that the use of music over long periods of time may come with increasing or long-term benefits. In terms of the anxiolytic effects specifically, the use of biomarkers such as cortisol or oxytocin may reveal potential biological mechanisms behind a music-sleep effect. This merits further investigation.

Whilst supporting the anecdotal idea that a key reason to select music for sleep is to aid relaxation, the survey identified for the first time a larger collection of motivators for using music when sleep is disturbed. The use of music as a distractor was a prominent theme, with distraction against thoughts (and particularly negative thoughts) a frequent comment that would benefit from further research. Negative thoughts are one of the main contributors to sleep loss in people with insomnia [60,61] and distraction of these thoughts was one of the main reasons reported for the use of music throughout the survey. We propose that the potential interaction between cognitive control of negative thoughts and music may be of significant importance for unearthing the pathways by which music aids in the production, maintenance, and enjoyment of sleep.

Our thematic analysis also revealed a number of other important reasons that people use music to aid sleep, which are not commonly discussed. Habitual use of music as a part of a user’s sleep hygiene was important to some respondents. This may be as part of a systematic regulation of a sleep hygiene routine, which is commonly utilized as a treatment option for individuals with insomnia [62]. It was also reported that masking external sounds, which can often lead to poor sleep quality, was a significant motivation for using music during sleep. More generally, there is a larger variety in the reported motivations for selecting music during sleep than was expected based on the existing literature. Future research should take this diversity into account when studying music as a sleep intervention option.

There are three important limitations to this study. In the first place and as already noted, we are unable to draw conclusions about the effectiveness of music on sleep physiology and underlying sleep mechanisms based on survey results alone. Research has shown that subjective and objective measures of sleep physiology are not always closely linked and hence the reported positive benefits of music on sleep may not be reflected in objective sleep measurements [46]. However, research using both objective and subjective sleep measures have shown improvements in both measures as a result of music [28], so this remains an open question and suggests that our survey is a useful starting place for future sleep studies looking at objective measures of music’s effectiveness as a sleep aid. The second limitation is that, due to the nature of the online survey, the subject matter and the methods of recruitment, we observed some sample bias in age, with a disproportionately high number of young respondents. However, we did see a broad range of participants with our youngest participants being 18 years old, and our oldest being 79 years old. The third and perhaps most important limitation is that the self-selected nature of the survey participants means that the survey is likely to biased towards music users, even though our data indicate that non-users were equally able to take part. This limitation means that the proportion of music users is likely to be over-estimated within the survey, though without any additional sources of information, it is impossible to know by how much. Including the question in a broader lifestyle survey might be one way to overcome this limitation in future work.

The need for a non-pharmaceutical, low cost sleep aid within our modern society is clear due to the economic, physical and psychological costs of sleep loss, which are increasingly widespread. Many people endure inadequate sleep on a regular basis and whilst pharmaceutical and over-the-counter options can provide some relief, many are ineffective and can lead to short term and chronic health-related side effects. Music’s potential for successful use in therapeutic and clinical settings makes it a viable, low cost, side-effect free option in the treatment of sleep loss. The results of this population survey suggest that many individuals are already intuitively using diverse types of music to fight sleep difficulties. Based on the cascade of potential factors that influence music choices that we have identified (body and brain mechanisms linked to anxiolytic properties of music, personal preference, demographic factors) and, in particular, the wide range of reasons we identified that drive music’s potential to improve sleep in different circumstances, future studies can investigate how various flexible and personalized music interventions can be developed to best target the various causes of sleep loss.

## Supporting information

S1 Data (XLSX)

## References

[1] Decker T. The Gull’s Hornbook. London; 1812.

[2] Royal Society for Public Health [Internet]. 2016 [cited 2017 Jan 1]. https://www.rsph.org.uk/

[3] CDC. Insufficient Sleep Is a Public Health Epidemic [Internet]. CDCFeatures: Data & Statistics. 2014. http://www.cdc.gov/features/dssleep/

[4] Chee MW, Chuah YM. Functional neuroimaging and behavioral correlates of capacity decline in visual short-term memory after sleep deprivation. Proc Natl Acad Sci U S A [Internet]. 2007/05/21. 2007;104(22):9487–92. https://www.ncbi.nlm.nih.gov/pubmed/17517619 10.1073/pnas.0610712104 17517619 PMC1874228

[5] Paterson JL, Dorrian J, Ferguson SA, Jay SM, Lamond N, Murphy PJ, et al Changes in structural aspects of mood during 39–66 h of sleep loss using matched controls. Appl Erg [Internet]. 2010/07/24. 2011;42(2):196–201. https://www.ncbi.nlm.nih.gov/pubmed/20659729 10.1016/j.apergo.2010.06.014 20659729

[6] Durmer JS, Dinges DF. Neurocognitive consequences of sleep deprivation. Semin Neurol [Internet]. 2005;25(1):117–29. https://www.ncbi.nlm.nih.gov/pubmed/15798944 10.1055/s-2005-867080 15798944

[7] Knutson KL, Van Cauter E. Associations between sleep loss and increased risk of obesity and diabetes. Ann N Y Acad Sci [Internet]. 2008;1129:287–304. https://www.ncbi.nlm.nih.gov/pubmed/18591489 10.1196/annals.1417.033 18591489 PMC4394987

[8] Spira AP, Chen-Edinboro LP, Wu MN, Yaffe K. Impact of sleep on the risk of cognitive decline and dementia. Curr Opin Psychiatry [Internet]. 2014;27(6):478–83. https://www.ncbi.nlm.nih.gov/pubmed/25188896 10.1097/YCO.0000000000000106 25188896 PMC4323377

[9] Parthasarathy S, Vasquez MM, Halonen M, Bootzin R, Quan SF, Martinez FD, et al Persistent insomnia is associated with mortality risk. Am J Med [Internet]. 2014/10/16. 2015;128(3):268–75.e2. https://www.ncbi.nlm.nih.gov/pubmed/25447616 10.1016/j.amjmed.2014.10.015 25447616 PMC4340773

[10] Yarrow K. The Sleep Industry: Why We’re Paying ig Bucks for Something That’s Free. Time [Internet]. 2013; http://business.time.com/2013/01/28/the-sleep-industry-why-were-paying-big-bucks-for-something-thats-free/

[11] Adams S. Sleeping Pill Cost to NHS Almost £50m. [Internet]. The Telegraph. 2012 [cited 2015 Sep 11]. http://www.telegraph.co.uk/news/health/news/9257191/Sleeping-pill-cost-to-NHS-almost-50m.html

[12] NHS Sleeping Pill Spend Leaps to £50m. The Guardian2. 2012.

[13] Barton L. Sleeping Pills: Britain’s Hidden Addiction [Internet]. The Guardian. 2012 [cited 2015 Sep 11]. http://www.theguardian.com/lifeandstyle/2012/aug/20/sleeping-pills-britains-hidden-addiction

[14] Ford ES, Wheaton AG, Cunningham TJ, Giles WH, Chapman DP, Croft JB. Trends in Outpatient Visits for Insomnia, Sleep Apnea, and Prescriptions for Sleep Medications among US Adults: Findings from the National Ambulatory Medical Care Survey 1999–2010. Sleep [Internet]. 2014;37(8):1283–93. http://www.ncbi.nlm.nih.gov/pubmed/25083008 10.5665/sleep.3914 25083008 PMC4096197

[15] Gershell L. Insomnia market. Nat Rev Drug Discov [Internet]. 2006;5(1):15–6. https://www.ncbi.nlm.nih.gov/pubmed/16485342 10.1038/nrd1932 16485342

[16] Degenhardt L, Darke S, Dillon P. GHB use among Australians: characteristics, use patterns and associated harm. Drug Alcohol Depend [Internet]. 2002;67(1):89–94. https://www.ncbi.nlm.nih.gov/pubmed/12062782 12062782 10.1016/s0376-8716(02)00017-0

[17] Koelsch S, Boehlig A, Hohenadel M, Nitsche I, Bauer K, Sack U. The impact of acute stress on hormones and cytokines, and how their recovery is affected by music-evoked positive mood. Sci Rep [Internet]. 2016/03/29. 2016;6:23008 https://www.ncbi.nlm.nih.gov/pubmed/27020850 10.1038/srep23008 27020850 PMC4810374

[18] Fancourt D, Williamon A, Carvalho LA, Steptoe A, Dow R, Lewis I. Singing modulates mood, stress, cortisol, cytokine and neuropeptide activity in cancer patients and carers. Ecancermedicalscience [Internet]. 2016/04/05. 2016;10:631 https://www.ncbi.nlm.nih.gov/pubmed/27170831 10.3332/ecancer.2016.631 27170831 PMC4854222

[19] Nilsson U. The anxiety- and pain-reducing effects of music interventions: a systematic review. AORN J [Internet]. 2008;87(4):780–807. https://www.ncbi.nlm.nih.gov/pubmed/18395022 10.1016/j.aorn.2007.09.013 18395022

[20] Fancourt D, Ockelford A, Belai A. The psychoneuroimmunological effects of music: a systematic review and a new model. Brain Behav Immun [Internet]. 2013/10/21. 2014;36:15–26. https://www.ncbi.nlm.nih.gov/pubmed/24157429 10.1016/j.bbi.2013.10.014 24157429

[21] Linnemann A, Ditzen B, Strahler J, Doerr JM, Nater UM. Music listening as a means of stress reduction in daily life. Psychoneuroendocrinology [Internet]. 2015/06/21. 2015;60:82–90. https://www.ncbi.nlm.nih.gov/pubmed/26142566 10.1016/j.psyneuen.2015.06.008 26142566

[22] Thoma M V, La Marca R, Brönnimann R, Finkel L, Ehlert U, Nater UM. The effect of music on the human stress response. PLoS One [Internet]. 2013/08/05. 2013;8(8):e70156 https://www.ncbi.nlm.nih.gov/pubmed/23940541 10.1371/journal.pone.0070156 23940541 PMC3734071

[23] Nilsson U. Soothing music can increase oxytocin levels during bed rest after open-heart surgery: a randomised control trial. J Clin Nurs [Internet]. 2009;18(15):2153–61. https://www.ncbi.nlm.nih.gov/pubmed/19583647 10.1111/j.1365-2702.2008.02718.x 19583647

[24] Chanda ML, Levitin DJ. The neurochemistry of music. Trends Cogn Sci [Internet]. 2013;17(4):179–93. https://www.ncbi.nlm.nih.gov/pubmed/23541122 10.1016/j.tics.2013.02.007 23541122

[25] Saarikallio S. Music as emotional self-regulation throughout adulthood. Psychol Music. 2011;39(3):307–27.

[26] Hernández-Ruiz E. Effect of music therapy on the anxiety levels and sleep patterns of abused women in shelters. J Music Ther. 2005;42:140–58. 15913391 10.1093/jmt/42.2.140

[27] Saarikallio S, Erkkila J. The role of music in adolescents’ mood regulation. Psychol Music. 2007;35(1):88–109.

[28] Chang ET, Lai HL, Chen PW, Hsieh YM, Lee LH. The effects of music on the sleep quality of adults with chronic insomnia using evidence from polysomnographic and self-reported analysis: a randomized control trial. Int J Nurs Stud [Internet]. 2012/04/10. 2012;49(8):921–30. https://www.ncbi.nlm.nih.gov/pubmed/22494532 10.1016/j.ijnurstu.2012.02.019 22494532

[29] Harmat L, Takács J, Bódizs R. Music improves sleep quality in students. J Adv Nurs [Internet]. 2008;62(3):327–35. https://www.ncbi.nlm.nih.gov/pubmed/18426457 10.1111/j.1365-2648.2008.04602.x 18426457

[30] Shum A, Taylor BJ, Thayala J, Chan MF. The effects of sedative music on sleep quality of older community-dwelling adults in Singapore. Complement Ther Med [Internet]. 2013/11/14. 2014;22(1):49–56. https://www.ncbi.nlm.nih.gov/pubmed/24559816 10.1016/j.ctim.2013.11.003 24559816

[31] Chen C-K, Pei Y-C, Chen N-H, Huang L-T, Chou S-W, Wu KP, et al Sedative Music Facilitates Deep Sleep in Young Adults. J Altern Complement Med. 2014; 10.1089/acm.2012.0050 23663079

[32] Jespersen K V, Koenig J, Jennum P, Vuust P. Music for insomnia in adults. Cochrane Database Syst Rev [Internet]. 2015/08/13. 2015;(8):CD010459 https://www.ncbi.nlm.nih.gov/pubmed/26270746 10.1002/14651858.CD010459.pub2 26270746 PMC9241357

[33] Lazic SE, Ogilvie RD. Lack of efficacy of music to improve sleep: A polysomnographic and quantitative EEG analysis. Int J Psychophysiol. 2007; 10.1016/j.ijpsycho.2006.10.004 17123654

[34] Qualtrics [Internet]. Provo, Utah, USA: Qualtrics; 2005. https://www.qualtrics.com

[35] Müllensiefen D, Gingras B, Musil J, Stewart L. The musicality of non-musicians: an index for assessing musical sophistication in the general population. PLoS One [Internet]. 2014/02/26. 2014;9(2):e89642 https://www.ncbi.nlm.nih.gov/pubmed/24586929 10.1371/journal.pone.0089642 24586929 PMC3935919

[36] Buysse DJ, Reynolds CF, Monk TH, Berman SR, Kupfer DJ. The Pittsburgh Sleep Quality Index: a new instrument for psychiatric practice and research. Psychiatry Res [Internet]. 1989;28(2):193–213. https://www.ncbi.nlm.nih.gov/pubmed/2748771 2748771 10.1016/0165-1781(89)90047-4

[37] Rentfrow PJ, Gosling SD. The do re mi’s of everyday life: the structure and personality correlates of music preferences. J Pers Soc Psychol [Internet]. 2003;84(6):1236–56. https://www.ncbi.nlm.nih.gov/pubmed/12793587 12793587 10.1037/0022-3514.84.6.1236

[38] Therneau T, Atkinson B, Ripley B, Ripley MB. rpart: Recursive Partitioning and Regression Trees. R Packag version 41–10 [Internet]. 2015; http://cran.ma.ic.ac.uk/web/packages/rpart/rpart.pdf%5Cnhttps://cran.r-project.org/web/packages/rpart/rpart.pdf

[39] Wickham H. ggplot2: Elegant Graphics for Data Analysis. [Internet]. Springer-Verlag New York; 2009 http://ggplot2.org

[40] Revelle W. psych: Procedures for Personality and Psychological Research. R Packag. 2016;

[41] Wickham H. Reshaping data with the reshape package. J Stat Softw. 2007;

[42] R Core Team. R Core Team (2014). R: A language and environment for statistical computing. R Found Stat Comput Vienna, Austria URL http://wwwR-project.org/.2014;R Foundation for Statistical Computing.

[43] Braun V, Clarke V. Using Thematic Analysis in Psychology. Qual Res Psychol. 2006;3(May 2015):77–101.

[44] Williamson VJ, Liikkanen LA, Jakubowski K, Stewart L. Sticky tunes: how do people react to involuntary musical imagery? PLoS One [Internet]. 2014/01/31. 2014;9(1):e86170 https://www.ncbi.nlm.nih.gov/pubmed/24497938 10.1371/journal.pone.0086170 24497938 PMC3908735

[45] Alessandri E, Williamson VJ, Eiholzer H, Williamon A. Beethoven recordings reviewed: A systematic method for mapping the content of music performance criticism. Front Psychol. 2015;6(FEB). 10.3389/fpsyg.2015.00057 PMC4330677 25741295

[46] Grandner MA, Kripke DF, Yoon I-Y, Youngstedt SD. Criterion validity of the Pittsburgh Sleep Quality Index: Investigation in a non-clinical sample. Sleep Biol Rhythms. 2006; 10.1111/j.1479-8425.2006.00207.x PMC3399671 22822303

[47] Mollayeva T, Thurairajah P, Burton K, Mollayeva S, Shapiro CM, Colantonio A. The Pittsburgh sleep quality index as a screening tool for sleep dysfunction in clinical and non-clinical samples: A systematic review and meta-analysis. Sleep Medicine Reviews. 2016. 10.1016/j.smrv.2015.01.009 26163057

[48] Strobl C, Malley J, Tutz G. An introduction to recursive partitioning: Rationale, application, and characteristics of classification and regression trees, bagging, and random forests. Psychol Methods [Internet]. 2009;14(4):323–48. http://psycnet.apa.org/journals/met/14/4/323.html%5Cnpapers3://publication/doi/10.1037/a0016973 10.1037/a0016973 19968396 PMC2927982

[49] Furihata R, Uchiyama M, Takahashi S, Konno C, Suzuki M, Osaki K, et al Self-help behaviors for sleep and depression: a Japanese nationwide general population survey. J Affect Disord [Internet]. 2010/10/12. 2011;130(1–2):75–82. https://www.ncbi.nlm.nih.gov/pubmed/20943273 20943273 10.1016/j.jad.2010.09.019

[50] Tan X, Yowler CJ, Super DM, Fratianne RB. The Interplay of Preference, Familiarity and Psychophysical Properties in Defining Relaxation Music. J Music Ther [Internet]. 2012;49(2):150–79. https://www.ncbi.nlm.nih.gov/pubmed/26753216 26753216 10.1093/jmt/49.2.150

[51] Lai H-L, Good M. Music improves sleep quality in older adults. J Adv Nurs. 2005; 10.1111/j.1365-2648.2004.03281.x 15660547

[52] Thompson B. Effects of Musical Tempo and Mode on Arousal, Mood, and Spatial Abilities. Music Percept Winter. 2002;20(2):151–71.

[53] Bauer AKR, Kreutz G, Herrmann CS. Individual musical tempo preference correlates with EEG beta rhythm. Psychophysiology. 2015;52(4):600–4. 10.1111/psyp.12375 25353087

[54] Istók E, Brattico E, Jacobsen T, Ritter A, Tervaniemi M. ‘I love Rock “n” Roll’—music genre preference modulates brain responses to music. Biol Psychol [Internet]. 2012/11/29. 2013;92(2):142–51. https://www.ncbi.nlm.nih.gov/pubmed/23201033 10.1016/j.biopsycho.2012.11.005 23201033

[55] Ohnishi T, Matsuda H, Asada T, Aruga M, Hirakata M, Nishikawa M, et al Functional anatomy of musical perception in musicians. Cereb Cortex [Internet]. 2001;11(8):754–60. https://www.ncbi.nlm.nih.gov/pubmed/11459765 11459765 10.1093/cercor/11.8.754

[56] Hunter PG, Glenn Schellenberg E, Stalinski SM. Liking and identifying emotionally expressive music: age and gender differences. J Exp Child Psychol [Internet]. 2011/04/29. 2011;110(1):80–93. https://www.ncbi.nlm.nih.gov/pubmed/21530980 10.1016/j.jecp.2011.04.001 21530980

[57] Dousty M, Daneshvar S, Haghjoo M. The effects of sedative music, arousal music, and silence on electrocardiography signals. J Electrocardiol [Internet]. 2011/02/24. 2011;44(3):396.e1–6. https://www.ncbi.nlm.nih.gov/pubmed/21353239 21353239 10.1016/j.jelectrocard.2011.01.005

[58] Huron D. Is music an evolutionary adaptation? Ann N Y Acad Sci [Internet]. 2001;930:43–61. https://www.ncbi.nlm.nih.gov/pubmed/11458859 11458859 10.1111/j.1749-6632.2001.tb05724.x

[59] MacDonald R. A., Mitchell L. A., Dillon T., Serpell M. G., Davies J. B., & Ashley EA. An Empirical Investigation of the Anxiolytic and Pain Reducing Effects of Music. 2Psychology Music. 2003;31(2):187–203.

[60] Kuisk L a, Bertelson a D, Walsh JK. Presleep cognitive hyperarousal and affect as factors in objective and subjective insomnia. Percept Mot Skills. 1989;69(3 Pt 2):1219–25. 10.2466/pms.1989.69.3f.1219 2622737

[61] Wicklow A, Espie CA. Intrusive thoughts and their relationship to actigraphic measurement of sleep: Towards a cognitive model of insomnia. Behav Res Ther. 2000;38(7):679–93. 10875190 10.1016/s0005-7967(99)00136-9

[62] Stepanski EJ, Wyatt JK. Use of sleep hygiene in the treatment of insomnia. Vol. 7, Sleep Medicine Reviews. 2003 p. 215–25. 10.1053/smrv.2001.0246 12927121

